# An audit of intensive care unit admission in a pediatric cardio-thoracic population in Enugu, Nigeria

**DOI:** 10.4314/pamj.v6i1.69077

**Published:** 2010-08-18

**Authors:** Ugochukwu Okafor, Jerome Azike

**Affiliations:** 1 University of Nigeria Teaching Hospital, Enugu, Nigeria; 2 Imo State University Teaching Hospital, Orlu, Imo State, Nigeria

**Keywords:** Audit, cardio-thoracic surgery, intensive care unit, anaesthesia, developing country,, Nigeria

## Abstract

Introduction: The study aimed to perform an audit of intensive care unit admissions in the paediatric cardio-thoracic population in Enugu, Nigeria and examine the challenges and outcome in this high risk group. Ways of improvement based on this study are suggested. Methods: The hospital records of consecutive postoperative pediatric cardiothoracic admissions to the multidisciplinary and cardiothoracic intensive care units of the University of Nigeria Teaching Hospital (UNTH) Enugu, Nigeria to determine their Intensive Care Unit management and outcome over a 2 year span -June 2002 to June 2004 were retrospectively reviewed. Data collected included patient demographics, diagnosis, duration of stay in the intensive care unit, therapeutic interventions and outcome. Results: There were a total of thirty consecutive postoperative paediatric admissions to the intensive care unit over the 2 year study period. The average age of the patients was 5.1 years with a range of 2 weeks to 13 years. Twelve patients had cardiac surgery with cardiopulmonary bypass (CPB), three patients had colon transplant, four patients had pericardiotomy/pericardicectomy, and five patients had diagnostic/therapeutic bronchoscopy. The remaining patients had the following surgeries, thoracotomy for repair of diaphragmatic hernia/decortications, delayed primary repair of esophageal atresia and gastrostomy. Two patients had excision of a cervical teratoma and cystic hygroma. The average duration of stay in the intensive care unit was 6.2 days. Ten patients (33%) received pressor agents for organ support. Five patients (17%) had mechanical ventilation, while twenty-five patients (83%) received oxygen therapy via intranasal cannula or endotracheal tube. Seven patients (23%) received blood transfusion in the ICU. There was a 66% survival rate with ten deaths. Conclusion: Paediatric cardio-thoracic services in Nigeria suffer from the problems of inadequate funding and manpower flight to better paying jobs. Government should invest in their people by introducing insurance schemes for cardiac patients. Training programmes for members of cardio-thoracic units in countries with advanced health care systems and hands on experience should be encouraged. Otherwise for a majority of children with heart disease, it will be a slow painful wait for the inevitable.

## Introduction

Pediatric cardiothoracic services are a big challenge in developing countries due to multifaceted problems. These include the enormous costs involved in countries with low Gross Domestic Product (GDP) like most countries in sub-Saharan Africa. This has largely resulted in poor infrastructure and with it, a large number of preventable deaths [1-3]; to these limitations can be added the flight of manpower to better paying jobs within and outside the country. The cost of subsidizing the management of the large number of children with congenital [1,2] and acquired cardiothoracic conditions has been proved beyond the means of most sub-Saharan African countries. The emergence of foundations like the Kanu Heart Foundation (KHF) founded by a famous soccer star, Nwankwo Kanu has stepped into this breach [4]. But it needs more than a few foundations to cater to these needs. Sub-Saharan Africa has other health problems like malaria and other infectious diseases such that those patients with cardiothoracic diseases fall down the prioritization list. Most of the recent literature on pediatric cardio-thoracic challenges has largely omitted West Africa and seems to focus more on East and Southern Africa without much literature from this region of about sixteen countries. In this study we look at the challenges, pattern and outcome for the post anaesthetic pediatric cardiothoracic patients managed in the intensive care unit of the University of Nigeria Teaching Hospital, Enugu Nigeria-Africa’s most populous nation.

## Methods

The hospital records of consecutive postoperative pediatric cardiothoracic admissions to the multidisciplinary and cardiothoracic intensive care units of the University of Nigeria Teaching Hospital (UNTH) Enugu, Nigeria were retrospectively reviewed to determine their ICU management and outcome over a 2 year span -June 2002 to June 2004. The following data were collected; patients demographics, diagnosis, therapeutic interventions and outcome. Children over 13 years were excluded from this study. Paediatric cardiac anaesthesia and surgery was provided by locally and internationally trained practitioners including a team from the United States. The cardiac intensive care unit is fairly well equipped with multi-channel monitors (pulse oximetry, invasive blood pressure and central venous pressure monitors, temperature, electrocardiography and capnography-recently), and modern ventilators.

## Results

There were a total of thirty consecutive postoperative paediatric admissions to the ICU over the 2 year study period. The average age of the patients was 5.1 years with a range of 2 weeks to 13 years. Twelve patients had cardiac surgery with cardiopulmonary bypass (CPB), three patients had colon transplant, four patients had pericardiotomy/pericardicectomy, and five patients had diagnostic/therapeutic bronchoscopy. The remaining patients had the following surgeries, thoracotomy for repair of diaphragmatic hernia/decortications, delayed primary repair of esophageal atresia and gastrostomy. Two patients had excision of a cervical teratoma and cystic hygroma. The average duration of stay in the intensive care unit was 6.2 days. Ten patients (33%) received pressor agents for organ support. Five patients (17%) had mechanical ventilation, while twenty-five patients (83%) received oxygen therapy via intranasal cannula or endotracheal tube. Seven patients (23%) received blood transfusion in the ICU. There was a 66% survival rate with ten deaths. The deaths were in six open heart surgery patients; two associated with rigid bronchoscopy and two others followed postoperative complications following excision of cystic hygroma and cervical teratoma.

## Discussion

In this study, the 30 pediatric cardiothoracic patients represented 27% of all cardiothoracic patients admitted to the ICU during the study period. Twelve pediatric patients (40%) had open heart surgery with cardio-pulmonary bypass, with a 50% percent success rate. This probably represented a small fraction of patients in need of open heart surgery as some die in infancy without medical assistance [4], and those that live are often too poor to pay the exorbitant operation fee. The elite usually receive treatment overseas. The cost of the disposal equipment and anaesthesia is usually less than 10% of the total fees. Since the hospital does not operate on a profit basis, government should help by subsidizing operation fees and/or providing needed materials for indigent patients.

Because our center is Nigeria’s major cardiothoracic center, the cardiac surgery theatre is well equipped with multi-channel monitors and invasive monitors used in cardiac patients. However, the hospital has been able to train only one cardiac anesthetist and two anesthetists/perfusionists over the past two decades. Both perfusionists have left for other jobs in centres without cardiac surgery units.

This might be reflected by the low priority given to pediatric cardiac surgery/anesthesia. The problems of malaria, HIV/AIDS, and other infectious diseases has sort of receded conditions like the pediatric anesthesia/surgery into the psychic landscape [5,6]. Anaesthesia was uneventful in the cardiac patients but there were three extubation failures which required re-intubation. The use of dexamethasone is believed to reduce the incidence of reintubation [7,8]. The mortality rate in this study is high compared to another study from the developing country of Guatemala [9]. The problems of pediatric cardiac surgery in the developing world has been documented in several studies [6,10-12] and to avoid a rehash of what has been written before, we will focus on the problems consequent on the outcome of this study. The first open heart surgery in Nigeria was performed in this center in 1973 [13,14]. Since then it has been regarded as Nigeria’s premier center for cardiothoracic surgery. The economic reversal in the 1980s and 1990s led to a flight of manpower overseas including some of the best surgeons and anaesthetists. The hospital has been able to train only one cardiac anesthetist and two anesthetists/perfusionists abroad over the past two decades. Both perfusionists have left for other jobs in centres without cardiac surgery units.

The 50% mortality rate among open heart surgery patients in this study can be partly attributed to that fact that most of the patients were already decompensated on presentation and preoperative evaluation may not have been optimal. The non-cardiac surgery patients had a better outcome. Two patients for bronchoscopy for removal of foreign bodies died postoperatively in the ICU following severe respiratory distress. The recent acquisition of fibreoptic bronchoscopes may reduce the mortality and morbidity in this group of patients. The two patients for excision of a cervical teratoma and cystic hygroma died as a result of surgical complications.

Pediatric cardiothoracic surgery in UNTH, Enugu needs an improvement with more equipment and personnel skills to manage cardio-thoracic cases. The government can assist hospitals by sponsoring members of cardiothoracic units to regular courses with hands on experience. This may be easier in the Indian Sub-continent and neighboring South Africa with their advanced cardio-thoracic practice, cheaper costs and more hands on experience [15]. If the Nigerian authorities have the will to invest in paediatric cardio-thoracic surgery, the results will be far-reaching. For example, Nigerian cardiac surgeons, anaesthetists and cardiologists practicing overseas should be encouraged and sponsored to return on short term basis to carry out cardio-thoracic surgery on indigent patients. Government investment in her people will help save lives and improve the nation’s Human Development Index as she seeks to join the comity of middle income nations.

## Conclusion

Paediatric cardio-thoracic services in Nigeria suffers from the myriad problems of inadequate funding, lack of sponsored training for members of cardio-thoracic units due to the apathy of administrators and manpower flight to better paying jobs. Overseas based Nigerian should make it a point of duty to form teams to perform surgeries on indigent patients on short term basis, while transferring knowledge to the home based anaesthetists and surgeons. Otherwise for a majority of children with heart disease, it will be a slow painful wait for the inevitable.

## Competing interests

There are no competing interests to declare.

## Authors’ contributions

UVO collected the data and analyzed it with significant intellectual input from HUE.
